# Protection of Gastrointestinal Mucosa from Acute Heavy Alcohol Consumption: The Effect of Berberine and Its Correlation with TLR2, 4/IL1β-TNFα Signaling

**DOI:** 10.1371/journal.pone.0134044

**Published:** 2015-07-30

**Authors:** Xin-Pei Wang, Fan Lei, Feng Du, Yu-Shuang Chai, Jing-Fei Jiang, Yu-Gang Wang, Xuan Yu, Xiao-Jin Yan, Dong-Ming Xing, Li-Jun Du

**Affiliations:** 1 MOE Key Laboratory of Protein Sciences, Laboratory of Molecular Pharmacology and Pharmaceutical Sciences, School of Life Sciences, Tsinghua University, Beijing, 100084, China; 2 School of Medicine, Tsinghua University, Beijing, 100084, China; 3 Department of Mathematics, Tulane University, New Orleans, LA, 70118, United States of America; 4 MD Anderson Cancer Center, University of Texas, Houston, Texas, 77030, United States of America; Oregon Health & Science University, UNITED STATES

## Abstract

The purpose of the present study is to confirm the protective effect of berberine (BBR) on gastrointestinal injury caused by acute heavy alcohol exposure, an effect that has not been reported previously. Our research details how BBR protects against gastrointestinal injuries from acute alcohol exposure using both *in vivo* and *in vitro* experiments. Acute high alcohol concentrations lead to obvious damage to the gastrointestinal mucosa, resulting in necrosis of the intestinal mucosa. Oral administration of BBR was able to significantly reduce this alcohol-induced damage, inhibit increases of alcohol-induced TNFα and IL-1β expression in gastrointestinal mucosa as well as their upstream signals TLR2 and TLR4, and regulate cytokines that modulate tight junctions. Alcohol consumption is a popular human social behavior worldwide, and the present study reports a comprehensive mechanism by which BBR protects against gastrointestinal injuries from alcohol stress, providing people with a novel application of BBR.

## Introduction

The Chinese medical herb, *Coptidis Rhizoma*, has a long history of clinic use and use as a food supplement. In ancient Chinese medical literature, *Coptidis Rhizoma* was originally recorded for its applications in the treatment of gastrointestinal dysfunction, including diarrhea, dysentery, and inflammation. Berberine (BBR) is the most abundant active natural compound in *Coptidis Rhizoma* and plays a major role in the pharmacological effects of *Coptidis Rhizoma*, including anti-cancer, anti-stroke, anti-diabetes and anti-hyperlipidemia effects [1–7]. BBR was recently reported to exhibit novel anti-inflammatory and anti-oxidant properties as well as the ability to inhibit gene transcription [8–11]. Recent studies reported the ability of BBR to maintain the junctions between intestinal mucosa [12–14], and the efflux pump in the small intestine mucosa can promote highly regional distribution of BBR in the gastrointestinal epithelia [15–19]. These findings indicate a potential application of BBR in the prohibition of gastrointestinal injury by excessive alcohol use. Alcohol consumption is a common social behavior worldwide. Alcohol use is a part of the culture and daily life of more than 2 billion people worldwide [20], and can play an essential role in business and social activities. After alcohol drinking, especially acute and extensive consumption, alcohol stimulates the gastro-intestinal tract and causes stress to the gastrointestinal mucosa, inducing gastrointestinal bleeding, inflammatory damage, and ulcers due to the upregulation of pro-inflammatory cytokines, IL-1β, IL6 and TNFα [21–23].

Based on prior studies, we hypothesized and tested the potential application of BBR in protecting against alcohol stress-induced gastrointestinal injuries. Both *in vivo* and *in vitro* experimental models of acute alcohol exposure were used to evaluate the effect of BBR on alcohol injury. The pro-inflammatory cytokines TNFα and IL-1β and the TLR2, TLR4, and NOD2 innate immunity signaling pathways were determined to be dynamically involved in gastrointestinal alcohol injury. BBR could effectively antagonize the regulation of the proinflammatory cytokine profile and alter innate immunity signaling downstream of acute extensive alcohol stress through direct effects on gene transcription. Furthermore, BBR antagonized elevations of blood alcohol by prohibiting alcohol absorption and up-regulating ADH (alcohol dehydrogenase) activity to accelerate the metabolism of absorbed alcohol.

## Methods and Materials

### Experimental animals, drugs and chemicals

Male ICR mice weighing 18–22 g were purchased from Vital River Laboratories (Beijing, China). The animals were housed in temperature- and humidity-controlled rooms, kept on a 12 h light/dark cycle and provided with unrestricted amounts of rodent chow and drinkable water. The laboratory animal facility was accredited by the AAALAC (Association for Assessment and Accreditation of Laboratory Animal Care International), and the IACUC (Institutional Animal Care and Use Committee) of Tsinghua University approved all animal protocols used in this study (Approval ID: 13-DLJ1).

Berberine hydrochloride was purchased from the Beijing Shuanghe Pharmacy Co. Ltd. (Batch No. 110905) (Beijing, China). Berberine hydrochloride standards were purchased from the National Institutes for Food and Drug Control (Beijing, China) (Batch No. 110713–200609). Isolated alcohol (analytical grade) was purchased from Beijing Chemical Plant (Beijing, China).

### Dosage and use of BBR and alcohol

Based on our prior experiments, BBR was administered orally at 75,150, or 300 mg/kg in saline vehicle. Three hundred milligrams/kilogram was far from the toxic dosage, and 150 mg/kg is the equivalent dose of adults in clinic [15].

Sixty percent alcohol (V/V) was employed for the experiments. Alcohol and normal saline was mixed to a final concentration of 60%. Based on the preliminary experiments, the volume for oral administration was 15 ml/kg (approximately 7.103 g/kg), and the samples were collected 2 hours after alcohol oral administration. BBR was administered orally 1 hour prior to alcohol consumption.

### Experimental procedures *in vivo*

#### Absorption of alcohol and enzymatic activity

ICR mice were divided into five groups randomly (six mice in each group). BBR was administered orally to animals in three different groups. One hour later, 60% alcohol (0.15 ml/10 g body weight) was administered orally. The normal group was administered saline only, and the model group was administered alcohol only. After two hours, blood samples were collected through the supraorbital veins, and the serum was isolated by centrifugation (15000 rcf/g, 10min, 4°C). All samples were stored at -80°C for alcohol concentration and enzyme activity determination.

#### Histopathological examination and diagnosis of gastrointestinal mucosa

The mice were randomly separated into five groups as previously described. Each group was comprised of six mice. Two hours after the administration of alcohol, the mice were sacrificed for morphological examination. All stomach and intestinal tissues were separated for gene expression analysis and histological determination. The histopathological diagnoses were performed through hematoxylin-eosin (HE) staining by two different research scientists independently. The samples were semi-quantitatively scored according to Shackelford et al [24].

#### Blood alcohol concentration

Alcohol concentrations were measured in accordance with the reference values. The reaction system was as follows: 1.5M Tris-HCl buffer (pH 8.8)350 μl, NAD^+^ (10 mg/ml) 100 μl, ADH (200 U/ml) 10 μl and serum samples 1 μl. The samples were mixed, and the OD value was recorded using a biochemical analyzer (Biosine Bio-technology and science Inc., China). The tested wavelength was set at 340 nm. The alcohol concentration is expressed in mg/ml.

#### ADH activity determination

ADH activity was measured in accordance with the instructions of the ADH kit (Nanjing Jiancheng Bioengineering Institute, China).The OD value was recorded using a microplate reader (BioRad, Model 680, USA) at OD_450nm_. The enzyme activity is expressed in U/ml.

### Experimental procedures *in vitro*

#### Cell culture and cell viability assay

The Caco2 and 293Tcell lines were obtained from the Cell Culture Center of Chinese Academy of Medical Science (Beijing, China) and maintained at 37°C in a humidified incubator containing 5% CO_2_. The cytotoxicity of BBR and alcohol to Caco2 cells and 293T cells was evaluated by MTT assay performed according to the method described in reference [25].

#### Alcohol-induced responses in the Caco2 cell line

2 μg/ml BBR (saline used as vehicle) was added one hour before acute alcohol exposure, and the concentration was maintained throughout the experiment. Two hours after alcohol administration, protein and RNA samples were extracted.

#### Promoter-GFP plasmid construction and transfection

The TLR2-pEGFPN1, NOD2-pEGFPN1 and pEGFP-N1 plasmids were kindly provided by Dr. Xiao-Jin Yan and Professor Ye-Guang Chen, School of Life Science, Tsinghua University. The TLR4 gene promoter was obtained from the mouse genome by PCR. Mouse genomic DNA was extracted from mouse liver homogenate (TIANamp Genomic DNA Kit, Tiangen Biotech, China). The CMV promoter of the pEGFP-N1 plasmid was replaced by the TLR4 promoter. GFP expression driven by the TLR2, TLR4 or NOD2 promoter was determined after transfection into the 293T cell line. The primer sequences of the TLR4 promoter were as follows: sense: 5’- AGAACAATGAAGGGACCCAGTC -3’ and antisense: 5’- GGGATTCAAGCTTCCTGGTGT -3’, generating a 1835-bp DNA fragment. The primer sequences of GFP were as follows: 5’- GCAGAAGAACGGCATCAAGG -3’ and antisense: 5’- CGGACTGGGTGCTCAGGTAG -3’.

### Real time PCR and western blot

mRNA and protein determination was performed using q-PCR and western blot (WB) assay as described in detail previously [26]. For real-time PCR, all primer sequences were designed by NCBI GenBank and produced by Sangon Biotechnology Ltd. (Shanghai, China) (S1 Table and S2 Table). For western blot analysis, primary antibodies against NOD2 (rabbit monoclonal antibody [EPR16252], ab197030), TNFα (mouse monoclonal antibody [52B83], ab1793), IL-1β (rabbit polyclonal antibody, ab9722), TLR2 (mouse monoclonal antibody [T2.5], ab16894), TLR4 (mouse monoclonal antibody [76B357.1], ab22048), Occludin (rabbit polyclonal antibody, ab64482) and claudin4 (rabbit polyclonal antibody, ab15104) were purchased from Abcam (UK). Secondary antibodies of goat anti-mouse IgG-HRP (sc-2005) and goat anti-rabbit (sc-2004) IgG-HRP were purchased from Santa Cruz (USA). The targeted proteins were and visualized with the Super Signal West Femto Chemiluminescent Substrate (Thermo scientific pierce) and the intensity of visualized bands were analyzed by using Quantity One software (Bio-rad). β-actin was used as an internal control. Data were expressed by the ratio to β-actin.

### Data analysis

Data are expressed as the mean ± S.D. Data were statistically analyzed using Kruskal–Wallis test. The test was performed using R software (USA). The non parametric Mann-Whitney U Test between two groups was performed after Kruskal–Wallis test. P values below 0.05 were considered statistically significant.

## Results

### Mouse gastrointestinal mucosal morphology and plasma alcohol concentration and ADH activity

**Fig 1:**
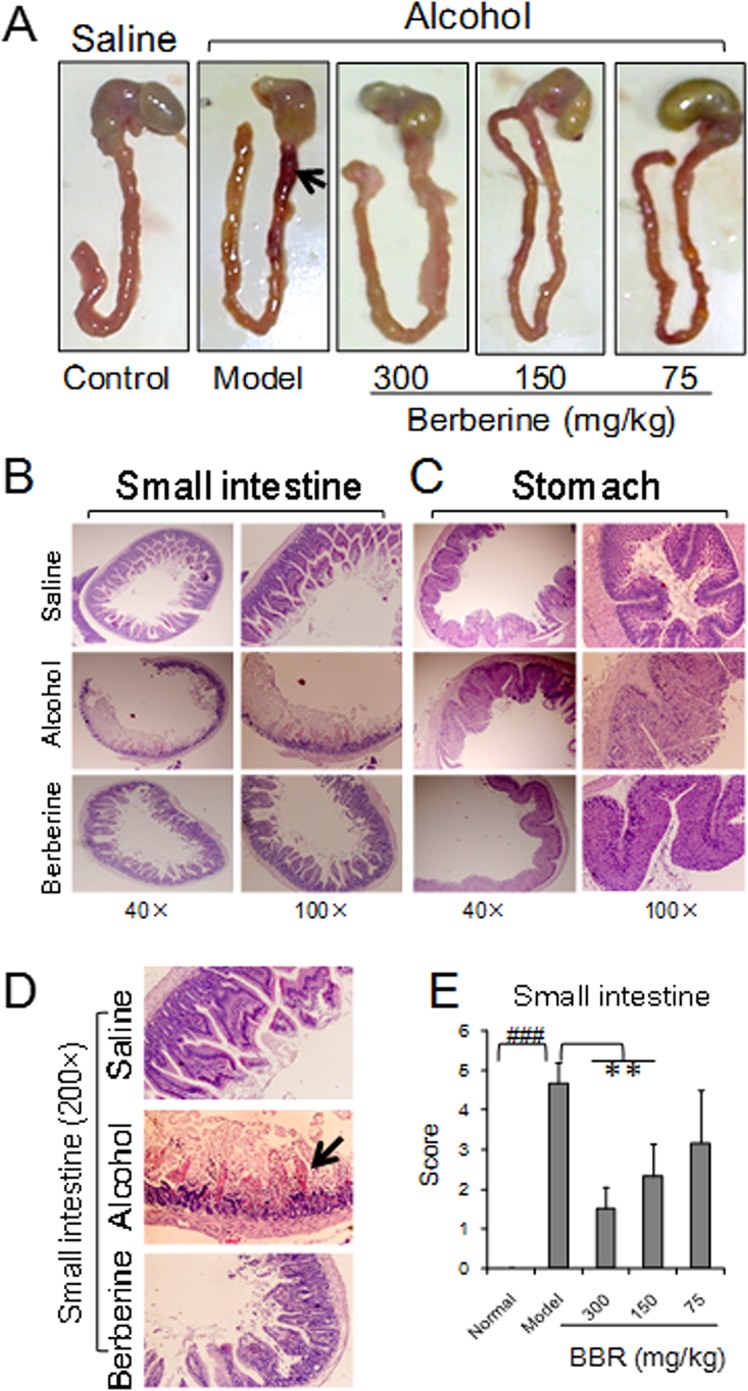
General observation and morphology of gastro-intestines after acute alcohol exposure (H.E. staining). Berberine (BBR) was administered at three different doses of 75, 150 and 300 mg/kg. A 60% alcohol was employed. The alcohol was administered at dose of 0.15 ml/10g body weight. The arrows indicate congestive necrosis places. (A): Observation of stomach and small intestines. Intestinal congestion occurs in the duodenum in model mouse. (B): Morphology of small intestines after alcohol administration. In the mouse model, small intestinal mucosa appears necrosis. BBR could prevent alcohol injury from the intestines. (C): Morphology of stomach after alcohol exposure. (D): Mucosa of small intestines (magnified 200 times). Alcohol causes gastric mucosal injury, edema with light staining. (E): Statistical score of the histopathological diagnoses for small intestines injury after alcohol consumption. Kruskal-Wallis chi-squared = 24.0696, df = 4, *P* = 7.735e-05. Data are expressed as the mean ± S.D. from six different mice. ###, *vs*. normal mice, *P* < 0.001. **, *vs*. model mice, *P* < 0.01.

Two hours after 60% alcohol administration, the alcohol-induced pathological damage was observed as a congestive and dark red appearance in the duodenum compared with that of normal mice. High-dose BBR (300mg/kg) effectively inhibited the alcohol-induced morphological changes of the duodenum (Fig 1A). By light microscopy, congestion, edema, necrosis and shedding of the mucosa from duodenum was observed in alcohol-treated mice (Fig 1B and 1D). BBR was able to effectively antagonize the alcohol-induced pathological changes in the duodenum, which was indistinguishable in morphology from the saline group. Unlike the small intestine, the gastric mucosa exhibited minor pathological changes among the different groups (Fig 1C). It has previously been reported that 100% alcohol can cause erosion in rat stomach mucosa and can up-regulate the mRNA expression of c-fos, c-jun and HSP70 in the damaged epithelium; however, the necrosis in the stomach was less severe than in the small intestine[27], suggesting that the small intestines were more sensitive to alcohol damage.

**Fig 2:**
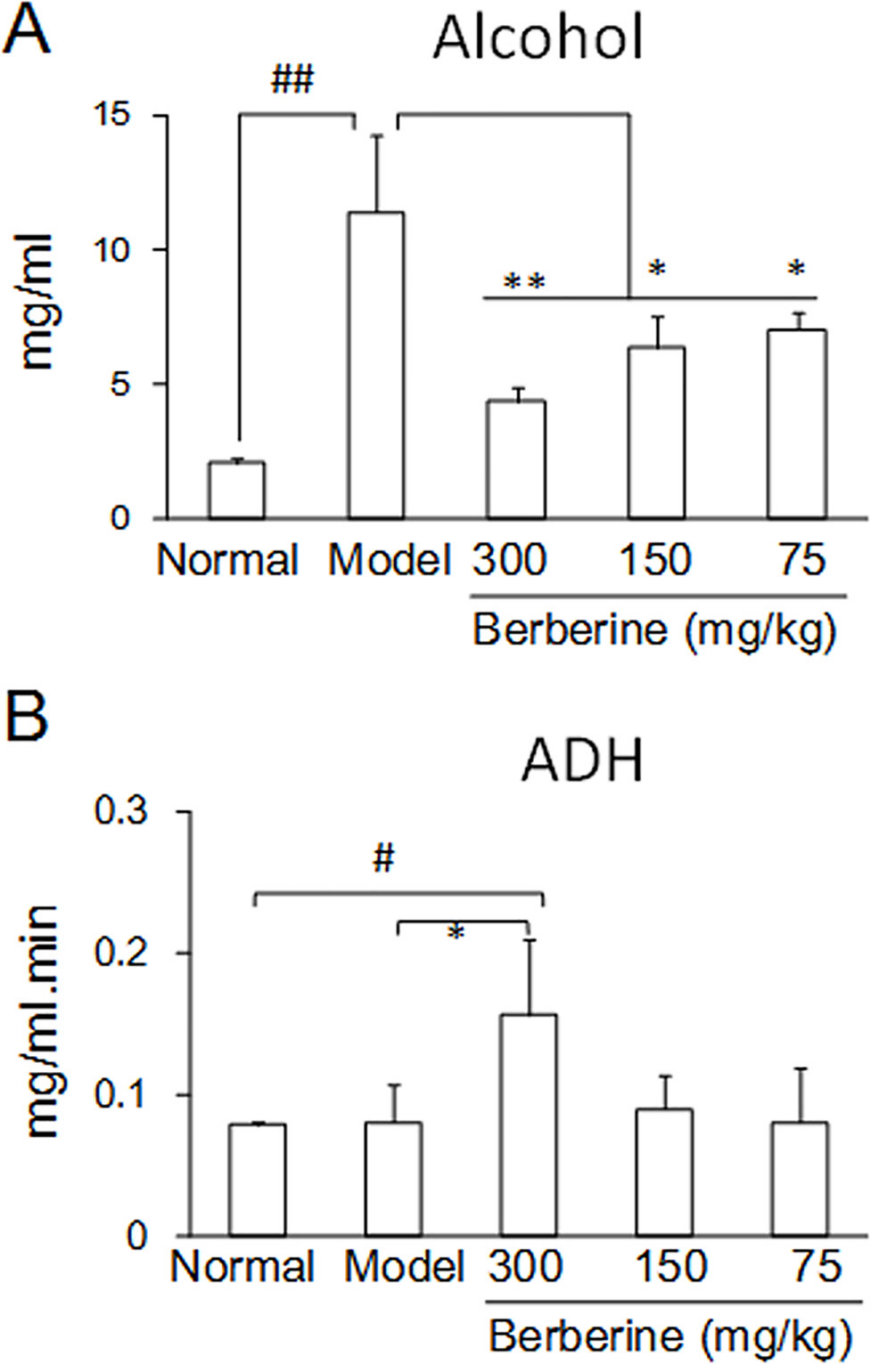
The concentration of alcohol and activity of ADH enzyme in blood of mice. (A): Alcohol, Kruskal-Wallis chi-squared = 26.6366, df = 4, *P* = 2.354e-05. (B): ADH. Kruskal-Wallis chi-squared = 14.0461, df = 4, *P* = 0.007149. Data are expressed as the mean ± S.D. from six different mice. #, ## *vs*. normal mice, *P* < 0.05, *P* < 0.01. *, ** *vs*. model mice, *P* < 0.05, *P* < 0.01.

The blood alcohol concentration (BAC) after alcohol consumption is the major factor causing drunkenness and body damage. Pretreatment with BBR before alcohol administration significantly reduced the BAC in mice even at the lower doses (Fig 2A). Furthermore, the plasma ADH activity was remarkably increased in the group treated with high-dose BBR, suggesting that in addition to the protective duodenum effect, BBR could protect mice from alcohol injury through decreased blood alcohol concentration and enhanced metabolism of alcohol by increased ADH activity (Fig 2B).

### Effect of BBR on the pro-inflammatory cytokines profile and pattern recognition receptors in mouse stomach after acute alcohol exposure

**Fig 3:**
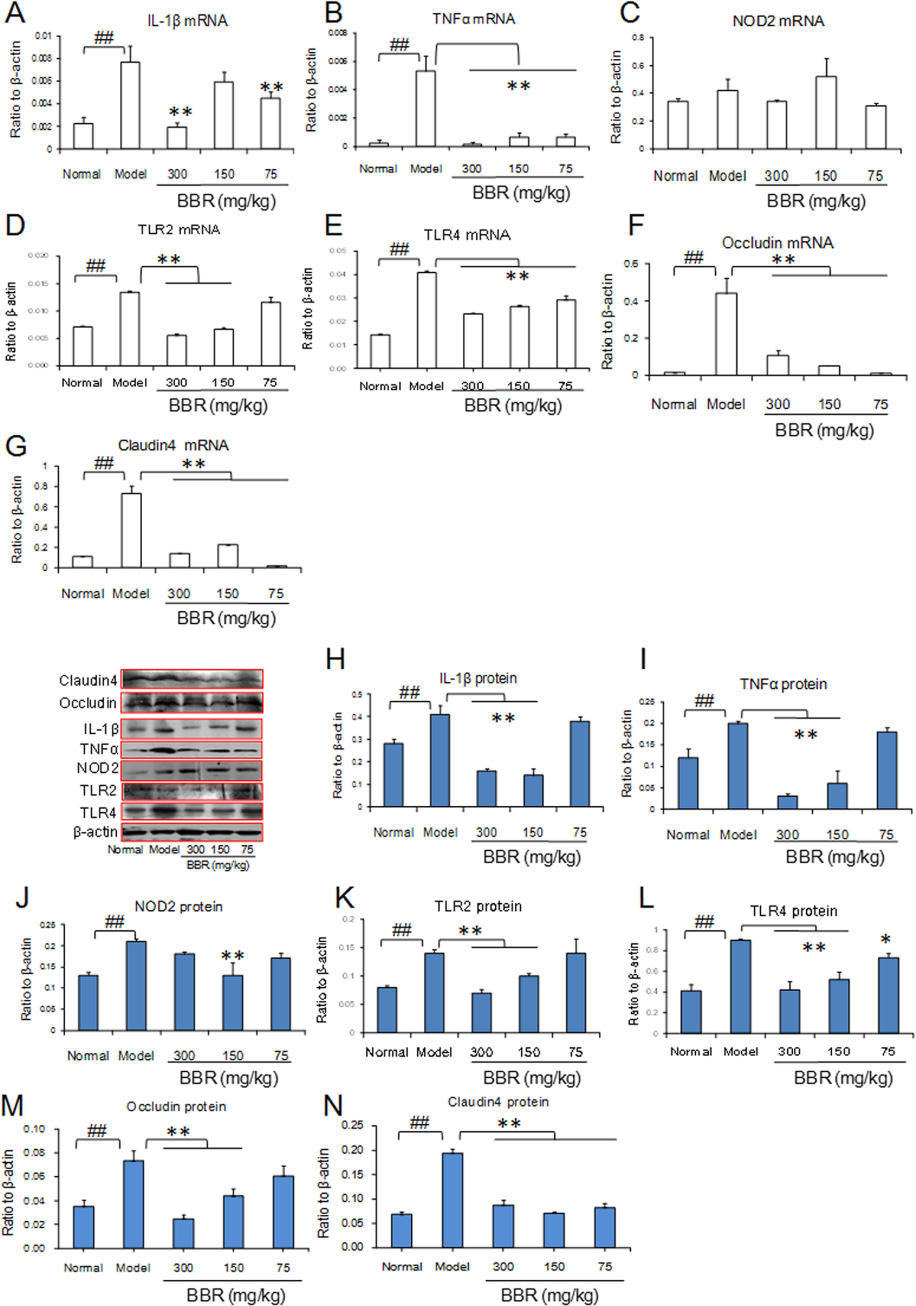
The expressions of inflammatory cytokines of mouse stomach after acute alcohol exposure. The concentration of alcohol was 60%. (A–G): mRNA expressions using real time PCR assay. (H–N): The expressions of protein using western blot assay. Berberine (BBR) was administered at three different doses of 75,150 and 300 mg/kg. Data are expressed as the mean ± S.D. from six different mice. ## *vs*. normal mice, *P* < 0.01. *, ** *vs*. model mice, *P* < 0.05, *P* < 0.01.

To understand the inflammatory response and abnormal expression of pattern recognition receptors (e.g., Toll-like receptors (TLRs) and NOD2) accompanying the ethanol-induced gastrointestinal mucosal injury, we studied the expression of TLR2, TLR4 and NOD2 and their down-stream effectors TNFα and IL1-β in mouse stomachs after acute alcohol exposure by q-PCR and western blot. The results revealed that acute alcohol exposure could significantly up-regulate the transcription and protein level of TNFα, IL1-β, TLR2, and TLR4, and these alcohol-dependent enhancements were antagonized by BBR administration (Fig 3). Only one BBR dose group exhibited decreased expression of NOD2 protein as well as consistent q-PCR results, suggesting that BBR exhibits a greater effect on TLR2 and TLR4 compared with NOD2 in the stomach mucosa. Occludin and claudin4 are major components of tight conjunctions in the gastrointestinal epithelium and act to regulate intestinal epithelial permeability. Acute high alcohol exposure can up-regulate occludin and claudin4 expression. However, alcohol-induced expression profile changes could be antagonized by BBR, which attenuated occludin and claudin4 expression (Fig 3F and 3G & 3M and 3N).

### Effect of BBR on the pro-inflammatory cytokines profile and TLRs and NOD2 in mouse small intestine after acute alcohol exposure

**Fig 4:**
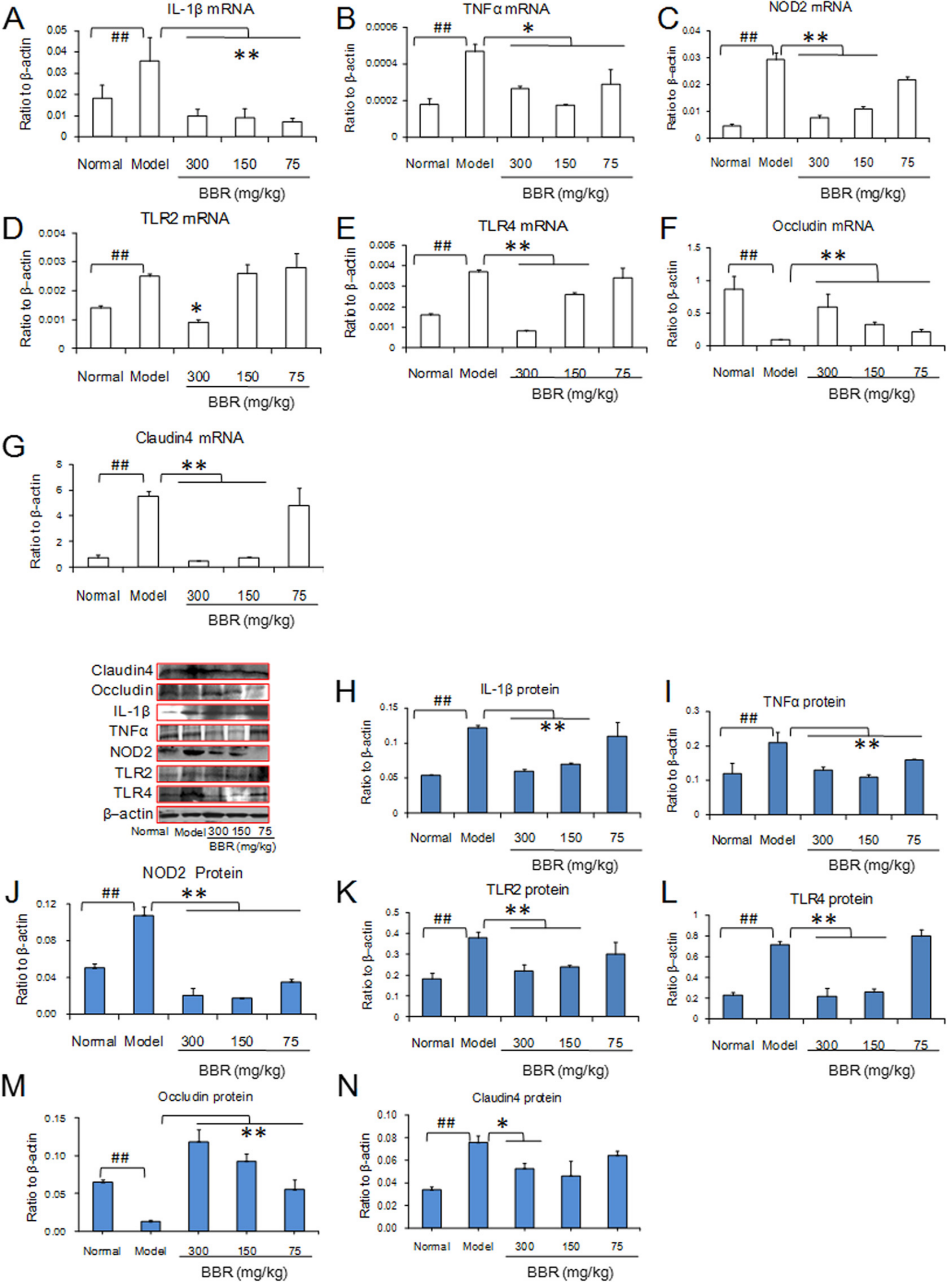
The expressions of inflammatory cytokines of mouse small intestines after acute alcohol exposure. The concentration of alcohol was 60%. (A–G): mRNA expressions using real time PCR assay. (H–N): The expressions of protein using western blot assay. Berberine (BBR) was administered at three different doses of 75,150 and 300 mg/kg. Data are expressed as the mean ± S.D. from six different mice. ## *vs*. normal mice, *P* < 0.01. *, ** *vs*. model mice, *P* < 0.05, *P* < 0.01.

Consistent with the studies in stomach, acute alcohol administration significantly increased the expressions of TNFα, IL1-β, TLR2, and TLR4 at the mRNA and protein level (Fig 4), and BBR dose-dependently inhibited alcohol-induced changes. However, inconsistent with NOD2 expression in the stomach, BBR inhibited the mRNA and protein upregulation of NOD2 caused by alcohol (Fig 4C& 4J), suggesting the greater sensitivity of the NOD2 response of the small intestinal mucosa to alcohol stress. The expression of other proteins was consistent with the mRNA results. In the small intestine, alcohol administration suppressed the expression of occludin but increased the expression of claudin4. BBR enhanced occludin expression and attenuated claudin4 expression (Fig 4F and 4G & 4M and 4N).

### Effect of BBR and alcohol on the Caco2 cell line *in vitro* after alcohol exposure

**Fig 5:**
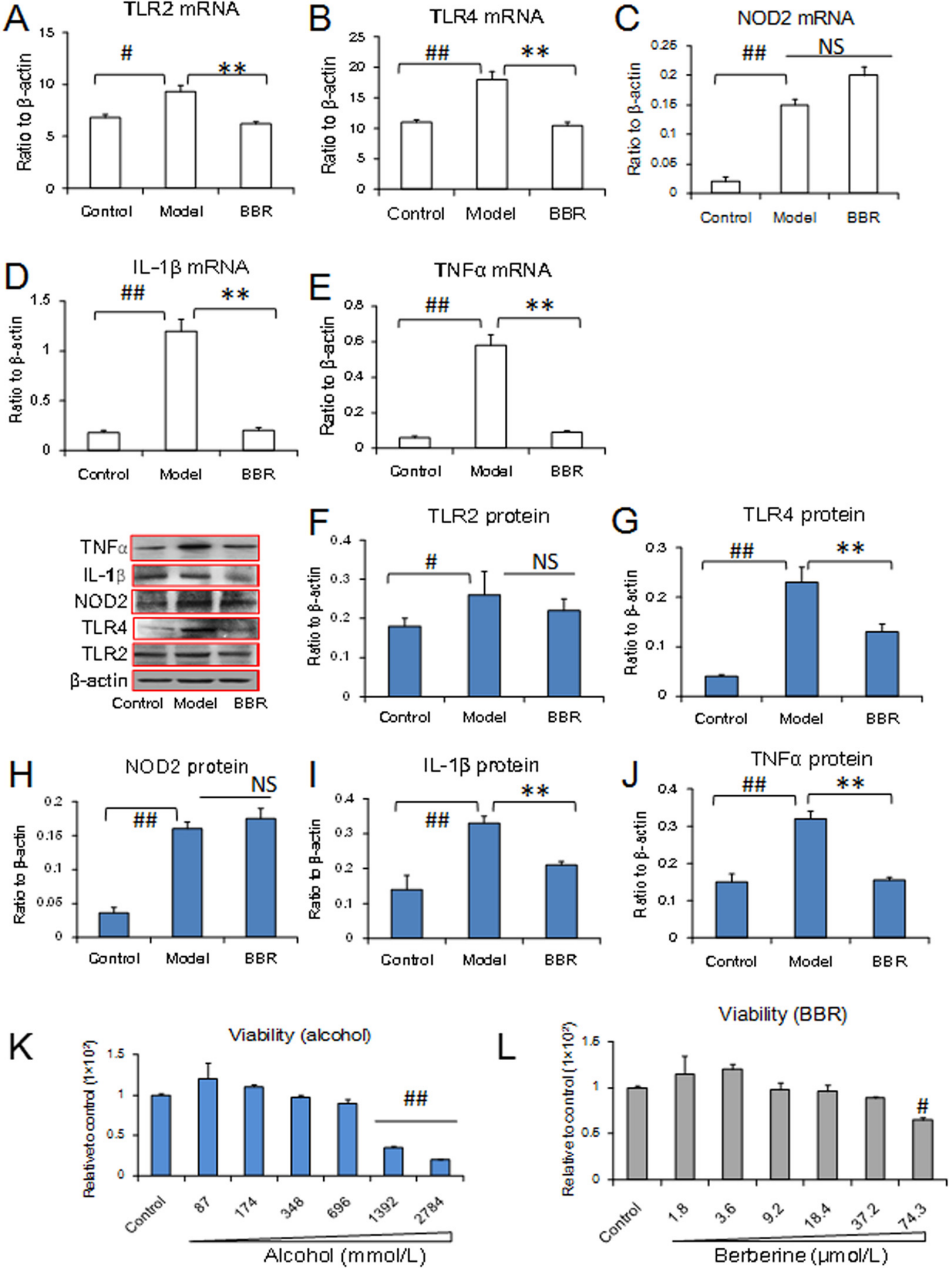
Expressions of inflammatory cytokines after berberine (BBR) on Caco2 cells injured by alcohol exposure *in vitro*. (A–E): mRNA expressions using real time PCR assay. (F–J): The expressions of protein using western blot assay. Alcohol was used at concentration of 348 mmol/L. BBR was administered at concentration of 5.95 μmol/L. (K): Cellular viability after alcohol exposure. (L): Cellular viability after BBR administration. Data are expressed as the mean ± S.D. from six independent experiments. #, ## *vs*. the control, *P* < 0.05, *P* < 0.01. ** *vs*. the model, *P* < 0.01.

To verify the effect of alcohol on small intestinal mucosal injury, we performed *in vitro* experiments using Caco2 cells, a human colon adenocarcinoma line exhibiting differentiation of small intestine epithelial cells [28]. Alcohol up-regulated the mRNA expression of the pro-inflammatory cytokines IL-1β and TNFα as well as the expression of the innate immune receptors TLR2, TLR4 and NOD2, which is consistent with the results observed in mice. BBR effectively decreased the expression of these genes, with the exception of NOD2 (Fig 5A–5E). In Caco2 cells, BBR exhibited little effect on NOD2 (Fig 5C). Similar results were observed at the level of protein expression (Fig 5F–5J). BBR down-regulated the protein expression of TLR2, but the trend did not reach statistical significance (Fig 5F). A 2% concentration of alcohol was administered (348 mmol/L) because the safety dosage for alcohol cytotoxicity was determined to be 696 mmol/L using the MTT assay (Fig 5K). BBR was administered at a dose of 2 μg/ml (5.95 μmol/L) in the experiments, which is far lower than the cytotoxic dosage of 37.2 μmol/L. The cytotoxicity of alcohol and BBR in Caco2 cells was measured using MTT assay (Fig 5L).

### Effect of BBR on TLR2, TLR4 and NOD2 promoters *in vitro*

**Fig 6:**
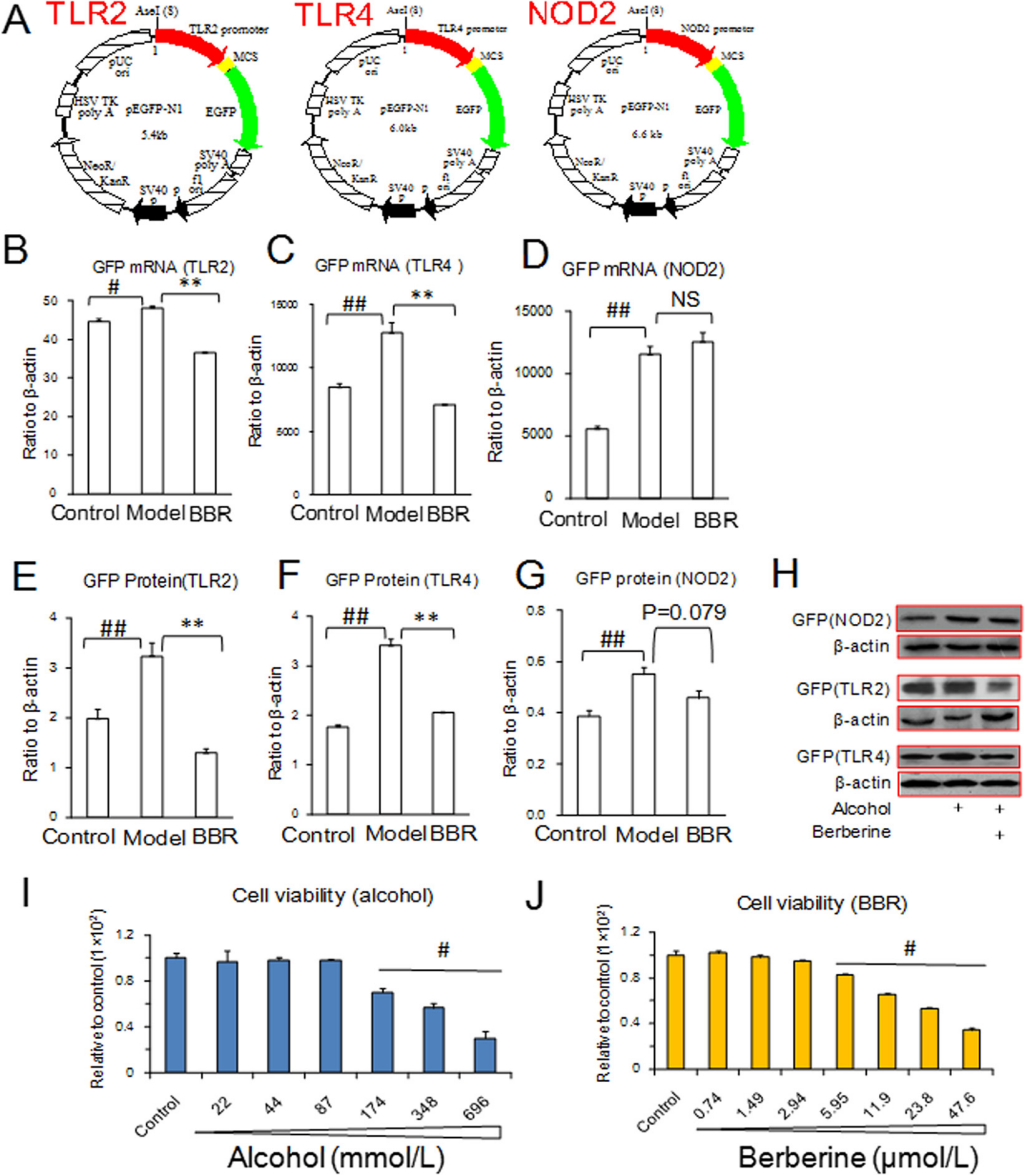
Effect of berberine (BBR) on the transcriptions of TLR2 and TLR4 on 293T cells. (A): Constructions of TLR2, TLR4 and NOD2 plasmids. (B–D): mRNA expression of GFP. (E–G): Protein expression of GFP promoted by TLR2, TLR4 and NOD2. (H): Lanes of protein expression detected using western blot assay. (I): Cellular viability after alcohol exposure. (J): Cellular viability after BBR administration. NS: no significance. Alcohol was used at concentration of 44 mmol/L. BBR was administered at concentration of 1.49 μmol/L. Data are expressed as the mean ± S.D. from three independent experiments. #, ## *vs*. the control, *P* < 0.05, *P* < 0.01. ** *vs*. the model, *P* < 0.01.

To acquire a more thorough understanding of the effect of BBR on TLR2, TLR4 and NOD2 expression, three promoter-driven expression plasmids were constructed (Fig 6A). Instead of CMV promoter, the TLR2, TLR4 or NOD2 promoters were used to drive the expression of green fluorescent protein (GFP). The expression of GFP was detected using q-PCR and WB assays. Alcohol promoted the mRNA expression of GFP downstream of the TLR2, TLR4 and NOD2 promoters. BBR was able to attenuate these stimulations and suppressed the up-regulated GFP mRNA expression driven by the TLR2 and TLR4 promoters (Fig 6B and 6C). However, BBR was unable to down-regulate GFP mRNA expression driven by the NOD2 promoter (Fig 6D). The protein expression of GFP in the TLR2 and TLR4 promoter plasmids were also inhibited by BBR, consistent with the results of mRNA expression (Fig 6E and 6F). Although the GFP protein expression of the NOD2 promoter plasmid exhibited a trend of down-regulation, it failed to reach statistical significance (P = 0.079) (Fig 6G). Therefore, we suggest that BBR suppresses alcohol-induced TLR2/TLR4 expression by interaction with their promoters. In these experiments, cells were treated with 44 mmol/L alcohol and 0.5 μg/ml (1.49 μmol /L) BBR, based on the results of a safety dose of alcohol of less than 87 mmol/L and a safety dose of BBR of less than 2.94 μmol/L in 293T cells (Fig 6I and 6J).

## Discussion

Alcoholic beverages have been consumed worldwide since the very beginning of recorded history [29]. Long-term or extensive drinking can cause harmful stresses to the central nervous system (CNS), cardiovascular system, immune system, liver, pancreas, gastrointestinal system, etc.[30, 31]. Thus, developing a strategy to alleviate alcohol consumption-related injury is pursued by many research groups worldwide.

In our preliminary experiments, alcohol induced the upregulation of inflammatory cytokines and the innate immunity response receptors TLR2, TLR4, and NOD2 in a dose-dependent manner and sixty percent alcohol could significantly alter the molecular profiles *in vivo* (Figures A and B in S1 File). Sixty percent alcohol-containing beverages are widely available on the market and are popular among alcohol consumers. Thus, 60% alcohol was selected to study the protective effect of BBR on alcohol-induced injury of the gastrointestinal system.

In some cases, extensive alcohol consumption is followed by inflammation [32, 33]. Alcohol- mediated inflammation signals are caused by the increased production of pro-inflammatory cytokines. In the present research, both *in vivo* and *in vitro* experiments revealed that alcohol could significantly promote the expression of the pro-inflammatory cytokine TNFα and IL1-β, which subsequently evokes inflammation. BBR antagonized alcohol-induced inflammation via suppressing the expression of pro-inflammatory cytokines.

Pattern recognition receptors act as the signaling molecules upstream of pro-inflammatory cytokines, such as IL-1β and TNFα [34–36]. The TLR4/MyD88 pathway had been confirmed as a target of alcohol-induced brain injury [37, 38]. TLR2 is an important mediator of inflammation in the airway epithelium induced by acute alcohol consumption [39].The present results revealed that alcohol could induce TLR2, TLR4 and NOD2 upregulation, and pre-treatment with BBR was able to antagonize the alcohol-enhanced expression of TLR2 and TLR4. Experimental results from recombinant plasmids studies indicated that gene transcription initiation is the direct target of BBR with respect to its antagonistic role on alcohol-regulated gene expression profiles. However, the antagonistic effect of BBR on alcohol-induced NOD2 signal pathway alternations was not obvious, indicating that the involvement of the NOD2 pathway in alcohol-induced inflammation differs somewhat from TLR2 and TLR4 and remains to be elucidated.

Occludin and claudins are the main proteins responsible for the gastrointestinal tract epithelial tight junctions (TJs) and the regulation of intestinal epithelial permeability [40, 41]. Occludin knockout mice exhibit decreased density and poor organization of the tight junctions in the intestinal mucosa as well as functional defects [42, 43]. The claudin super family plays critical roles in barrier formation and the selective permeability in tissues [44–48]. In the present study, acute heavy alcohol consumption resulted in an abnormal expression profile of occludin and claudin4 in the gastrointestinal mucosa, and BBR antagonizes these profile changes. Interestingly, we observed that alcohol could stimulate occludin downregulation and claudin4 upregulation in the small intestines, which has been previously reported [49].BBR was able to down-regulate claudin4 and up-regulate occludin, returning levels to homeostasis, suggesting that BBR’s potential effect on intestinal permeability altered alcohol-induced damage. Taken together, our results showed that alcohol could not only lead to an inflammatory reaction, but also affect mucosal permeability by modulating occludin and claudin4 expression. It was reported that TLR2 could influence the tight conjunction barrier in epidermal keratinocytes or cerebral endothelial cells [50–51]. BBR could inhibit the expression of TLR2, which would be involved in the mechanism of BBR on claudin4 and occludin. These putative mechanisms warrant further study.

Studies have demonstrated that inflammatory disorders induced elevated levels of IL-1β protein, followed by inhibition of the expression of occludin mRNA and enhanced intestinal mucosal permeability of TJs [52–54].According to these reports, high alcohol consumption can promote intestinal permeability [55].The present results revealed that acute high alcohol intake could cause injury of the mucosal layer of mouse small intestines, which was associated with up-regulated IL-1β expression and occludin suppression. BBR exhibited a consistent inhibitory effect on IL-1β expression, correlating with occludin expression up-regulation. TNFα is believed to be important in the incidence and development of inflammatory intestinal disease. Targeting TNFα can inhibit intestinal inflammation and improve intestinal permeability [56]. Our data demonstrates that high alcohol intake can increase the expression of TNFα in the duodenum and that BBR can reduce the upregulation of TNFα, suggesting that inhibition of TNFα expression is involved in the effects of BBR on acute heavy alcohol consumption-induced damages.

European men are reported to consume more than 0.72 g/kg alcohol daily, and women consume more than 0.65 g/kg [57]. On average, the daily normal alcohol consumption among Europeans is 60 g and 30 g for men and women, respectively. In addition, the maximum average daily intake of ethanol can reach 150 g [58–60]. Approximately 7.103 g/kg alcohol was administered in the present study, which is equivalent to a human dosage of 0.7805 g/kg [15]. Based on the average body weight of 70 kg, the total amount of alcohol scales to 54.638 g in the present study, which is close to the amount of alcohol reported earlier. BBR is able to prevent the damage inflicted at high doses, demonstrating that it could be used potentially as an effective therapy in clinical practice.

## Conclusions

In summary, this is the first report comprehensively demonstrating that pretreatment with BBR before acute alcohol consumption protects the gastrointestinal mucosa from alcohol injuries. The oral administration of BBR could effectively prevent gastrointestinal damage. The mechanism by which BBR conferred protective effects included the regulation of inflammatory cytokine profiles by directly targeting gene transcription, including the genes encoding TLR2 and TLR4. This work provides a reasonable therapeutic strategy to protect against gastrointestinal damage induced by acute heavy alcohol consumption.

## Supporting Information

S1 File Figure A. mRNA expressions of inflammatory cyctokines of mouse small intestines after oral administration of alcohol. Figure B. mRNA expressions of inflammatory cyctokines of mouse stomach after oral administration of alcohol. (DOCX)

S1 Table Primer sequence for q-PCR (mice). (DOCX)

S2 Table Primer sequence for q-PCR (Caco2 cells). (DOCX)
