# Supplementary material for: Protection of Gastrointestinal Mucosa from Acute Heavy Alcohol Consumption: The Effect of Berberine and Its Correlation with TLR2, 4/IL1β-TNFα Signaling
Source: PLoS One. 2015 Jul 30;10(7):e0134044. doi: 10.1371/journal.pone.0134044 (PMC4520689; doi:10.1371/journal.pone.0134044)
Supplement: S1 File — (DOCX) [file pone.0134044.s001.docx]

**Supporting information**

**Figure S1**

**Figure S1.** mRNA expressions of inflammatory cyctokines of mouse small intestines after oral administration of alcohol. The concentration of alcohol was 10, 20, 40 and 60% (V/V), respectively. The volume for oral administration was 0.15 ml/10 g bodyweight. Control mice were given with normal saline. (A): mRNA expressions of IL-1β. (B): mRNA expressions of TNFα. (C): mRNA expressions of TLR2. (D): mRNA expressions of TLR4. (E): mRNA expressions of NOD2. Data were expressed as mean ± S.D. from six different mice. ## *v.s*. control mice, *P* < 0.01.

**Figure S2**

**Figure S2.** mRNA expressions of inflammatory cyctokines of mouse stomach after oral administration of alcohol. The concentration of alcohol was 10, 20, 40 and 60% (V/V), respectively. The volume for oral administration was 0.15 ml/10 g bodyweight. Control mice were given with normal saline. (A): mRNA expressions of IL-1β. (B): mRNA expressions of TNFα. (C): mRNA expressions of TLR2. (D): mRNA expressions of TLR4. (E): mRNA expressions of NOD2. Data were expressed as mean ± S.D. from six different mice. ## *v.s*. control mice, *P* < 0.01.
