# Supplementary material for: Protection of Gastrointestinal Mucosa from Acute Heavy Alcohol Consumption: The Effect of Berberine and Its Correlation with TLR2, 4/IL1β-TNFα Signaling
Source: PLoS One. 2015 Jul 30;10(7):e0134044. doi: 10.1371/journal.pone.0134044 (PMC4520689; doi:10.1371/journal.pone.0134044)
Supplement: S1 Table — (DOCX) [file pone.0134044.s002.docx]

**Supporting information Tables**

Table S1

Table S1. Primer sequence for q-PCR (mice)

| **Gene** | **Sense** | **Anti-sense** | **Products (bp)** |
| --- | --- | --- | --- |
| NOD2  NM_145857 | TTGGAAAGGGGCCTGTGTATC | GGCTGCTGTCACTTCTTCTCA | 245 |
| TNFα  NM_013693.2 | GGGATCAAATCCAAGCCTGC | GTGAGGGTGGATGCTCAGTG | 223 |
| IL-1β  NM_008361 | CACACAAGGAAGTGCGTGTC | CACGTAGATGCACACCCAGA | 159 |
| TLR2  NM_011905.3 | TGATGGTGAAGGTTGGACGG | CCGGTGATGCAATTCGGATG | 164 |
| TLR4  NM_021297.2 | TCTCTGAACTTGAGAACATTTTGGG | TCAGTGTTCATGCTGACTGAAATAA | 150 |
| Occludin NM_008756.2 | AGGTGAGCACCTTGGGATTC | TTCAAAAGGCCTCACGGACA | 119 |
| Claudin4 NM_009903.2 | CAACTGCATGGAGGACGAGA | GGGTTGTAGAAGTCGCGGAT | 135 |
| MLCK  NM_139300.3 | AGCATGTCTGGCCAGTTACC | CTCCCTTCGAACTTGGCTGT | 194 |
| β-actin  NM_007393 | AGGCCACACAAATAGGGTCC | TTGTGGACACTGCCCCATTC | 158 |
