# Supplementary material for: Protection of Gastrointestinal Mucosa from Acute Heavy Alcohol Consumption: The Effect of Berberine and Its Correlation with TLR2, 4/IL1β-TNFα Signaling
Source: PLoS One. 2015 Jul 30;10(7):e0134044. doi: 10.1371/journal.pone.0134044 (PMC4520689; doi:10.1371/journal.pone.0134044)
Supplement: S2 Table — (DOCX) [file pone.0134044.s003.docx]

**Supporting information Tables**

Table S2

Table S2 Primer sequence for q-PCR (Caco2 cells)

| **Gene** | **Sense** | **Anti-sense** | **Products (bp)** |
| --- | --- | --- | --- |
| NOD2  NM_022162 | CACCGTCTGGAATAAGGGTACT | TTCATACTGGCTGACGAAACC | 229 |
| TNFα  NM_000594.3 | GACAAGCCTGTAGCCCATGT | CTCTGATGGCACCACCAACT | 132 |
| IL-1β  NM_000576 | AGCTACGAATCTCCGACCAC | CGTTATCCCATGTGTCGAAGAA | 186 |
| TLR2  NM_003264 | ATCCTCCAATCAGGCTTCTCT | GGACAGGTCAAGGCTTTTTACA | 118 |
| TLR4  NM_138557 | AGACCTGTCCCTGAACCCTAT | CGATGGACTTCTAAACCAGCCA | 147 |
| β-actin  NM_001101 | CATGTACGTTGCTATCCAGGC | CTCCTTAATGTCACGCACGAT | 250 |
